# HIV prevention is not all about HIV – using a discrete choice experiment among women to model how the uptake and effectiveness of HIV prevention products may also rely on pregnancy and STI protection

**DOI:** 10.1186/s12879-020-05399-4

**Published:** 2020-09-25

**Authors:** Peter Vickerman, Matthew Quaife, Maggie Kilbourne-Brook, Mercy Mvundura, Robyn Eakle, Fern Terris-Prestholt

**Affiliations:** 1 Population Health Sciences, University of Bristol, Bristol, BS8 2BN UK; 2 London School of Hygiene and Tropical Medicine, London, UK; 3 PATH, Seattle, USA

**Keywords:** HIV, South Africa, Women, Pre-exposure prophylaxis, Vaginal ring, Injectable long-lasting ARV agents

## Abstract

**Introduction:**

In sub-Saharan Africa, considerable HIV-burden exists among women. Anti-retroviral (ARV) based prevention products could decrease this burden, and their uptake could be increased if they also protect against pregnancy and sexually transmitted infections (STI).

**Methods:**

A discrete choice experiment (DCE) was undertaken in South Africa (2015) through a household survey of adult females (*n* = 158) and adolescent girls (*n* = 204) who self-reported HIV-negative status. The DCE was used to project the uptake (percentage using product) of oral pre-exposure prophylaxis (PrEP), vaginal rings, and injectable long-lasting ARV agents among these women, and how uptake could depend on whether these products protect against pregnancy or STI acquisition. Uptake estimates were used to model how each product could decrease a women’s HIV acquisition risk.

**Results:**

In adolescent women, there will be limited uptake (< 6% for any product) and impact (< 4% decrease in HIV acquisition risk) of new products unless they provide pregnancy protection, which could quadruple use and impact. Adult women have weaker preference for pregnancy protection, with moderate use (< 17% for each) and impact (< 14 percentage point decrease) if they only provide HIV protection. All women had highest preference for injectable ARVs, with oral PrEP having high preference if injectable ARVs are not available. Adult women will use the ring, but adolescent women will not. Importantly, even with three additional prevention products, all providing pregnancy and STI protection, > 14% of women will remain unprotected and > 31% of the baseline acquisition risk will remain.

**Conclusions:**

Incorporating multiple prevention components into new ARV-based prevention products may increase their uptake and impact among women.

## Introduction

In sub-Saharan Africa, considerable HIV burden exists amongst adolescent and adult women, accounting for 59% of new infections among adults in 2018 [1, 2]. Young women (15–24 years) in sub-Saharan Africa are more than twice as likely to acquire HIV than males of the same age [2]. Until recently, the only female controlled HIV prevention option was the female condom, which has limited availability and uptake [3, 4]. Otherwise, the male condom needs active participation of the male partner which can be problematic, particularly in casual relationships where power imbalances can make it difficult for young women to ensure that a condom is used [5, 6].

Biomedical HIV prevention products emerged after the HPTN 052 trial found that antiretroviral therapy (ART) dramatically reduced the infectiousness of HIV positive persons [7]. Although effective if used adherently, HIV treatment is not a panacea. For HIV-negative individuals in sero-discordant relationships to be protected, they must rely on their partner adhering to treatment, something that is not always achieved [8, 9]. For this reason, HIV treatment cannot be seen as an effective individual-level prevention method except in long-term relationships.

Oral pre-exposure prophylaxis (PrEP) is efficacious at reducing the risk of HIV acquisition amongst women when used adherently, as shown by a systematic review of trial data from 2016 [10]. However, trials have shown that young women can find it difficult to achieve this protection, with randomised controlled trials from sub-Saharan Africa showing low adherence and retention and, ultimately, no reduction in HIV acquisition risk amongst female users [11, 12]. Although recent demonstration projects among adolescent girls and young women in sub-Saharan Africa have shown similar difficulties in retention, higher adherence has been achieved [13]. Despite the efficacy of oral PrEP, there is an increasing realisation that multiple prevention options are needed to meet the varied lifestyles of potential users [14].

Preferences for products may vary by population group, and a combination approach may be needed to fulfil a women’s varied needs [15, 16]. In addition to PrEP, which is now available in South Africa, a number of longer-lasting products are in development [17, 18], with some likely becoming available in the next 5 years. These products may reduce the adherence and retention issues that exist for oral PrEP, which either must be taken daily or before and after sex. Longer-lasting products include the dapivirine vaginal ring that trials have shown can be effective at preventing HIV transmission [19], and injectable long-acting antiretroviral (ARV) agents which are currently in trial [20]. Unfortunately, in both existing trials of vaginal rings, younger women (18–21 years) had low adherence and efficacy [21, 22], although encouraging new data from open label extensions to these trials suggest higher efficacy [23]. The first trial of long-acting, injectable PrEP has also recently shown efficacy in men who have sex with men [24].

Models can be useful for projecting the impact of new prevention products [25–28]. However, before a product is used in a real-life setting it is difficult to predict how the product will be used, and how it will affect the use of other products. Indeed, even if a product has been evaluated in a trial setting, it is still hard to understand how it will be used in real-life. On one hand, it may be used more than in the trial because it has demonstrated efficacy, or conversely it may be used less because there will be less follow-up to ensure adherence compared to during a trial.

Trials also tell us little about how variations in the characteristics of different products may affect their level of use. For instance, a product which offers additional benefit, perhaps through higher HIV protection, protection against sexually transmitted infections (STI) or contraceptive properties, may have higher uptake and use. It is possible that new or existing products could incorporate these characteristics [29], and through doing so may achieve higher uptake and HIV prevention impact. For example, regular users of contraceptive products may not value HIV protection enough to take daily PrEP, but if HIV protection was built into a contraceptive product, considerable additional impact could be achieved [30]. Understanding what people value about potential prevention methods could make prevention products more responsive to end-user needs.

**Fig. 1:**
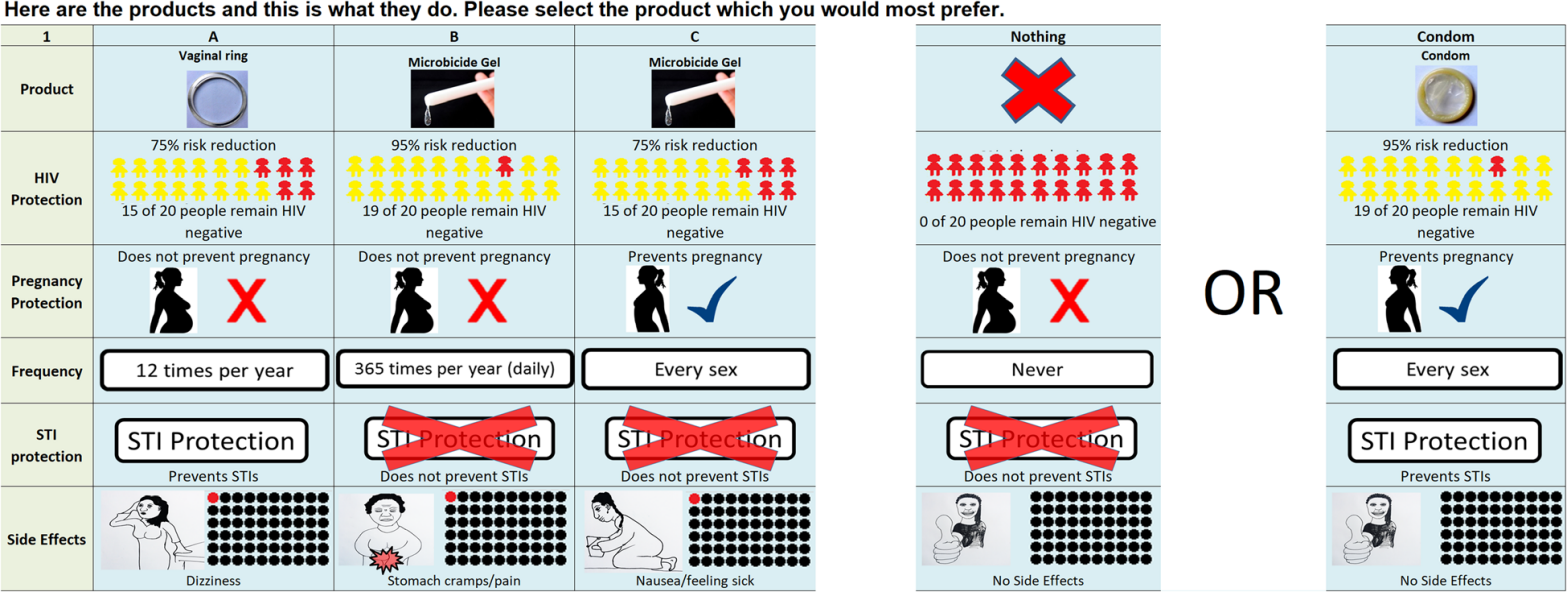
Example of a DCE task. Attributes shown in figure are just for illustration, and show that DCE’s consider options that are much wider than the product attributes given in Table 2

Evaluating how different product characteristics may impact demand for, or choices between products is challenging before they are introduced, particularly with no comparable products on the market. Economic theory suggests that consumer’s choices give insights into their underlying preferences, and the field of choice modelling has advanced to explore how choice data can be used to explore what people value [31–33]. Where no existing choice data exist to explore “revealed” preferences, i.e. observed use behaviour, one option is to elicit “stated” preferences. This can be done through a discrete choice experiment (DCE) where survey participants choose between hypothetical alternatives, each representing a specific product or service that is described by a number of more and less desirable attributes. Respondents are presented with a series of such choices, normally 8–10. An example of a DCE task is displayed in Fig. 1. By analysing the trade-offs respondents make, researchers can quantitatively explore what drives individual decision-making and the relative strength of preferences. DCEs are becoming increasingly popular in health services research [33–35].

In this analysis, we develop a previously published model [30, 36] to project the uptake of new and future products amongst young and adult women. We build on our published cost-effectiveness analysis [36] to look in detail at how the uptake of existing and new prevention products may be influenced by what products are already available, the prevention characteristics of those products, and the different preferences of adolescent and adult women. We also consider how the addition of these new products may reduce the prevention gap amongst adolescent and adult women, and how their additional prevention characteristics may contribute to this.

## Methods

We used a DCE to project the uptake of oral PrEP, which is now being rolled out in South Africa, and two other ARV-based HIV prevention products in the order that they are likely to become available on the market (Table 1): vaginal ring and injectable long-lasting ARV agents. We assessed how each product will affect the uptake of existing products, including the male condom, and how the characteristics of that product in terms of pregnancy and STI protection will affect its uptake. We assumed a **baseline** where only the male condom is available, with HIV efficacy of 85% (66–94% in uncertainty analysis) [37, 38] as well as STI and pregnancy protection, but only used in some sex acts. We then consider oral PrEP with just HIV efficacy, which is currently being introduced (**phase 1**). For oral PrEP, we assumed an HIV efficacy of 61% (40–75% in uncertainty analysis) as found among adherent women in a recent meta-analysis [10]. We then assume that the vaginal ring is introduced (**phase 2**) with HIV efficacy of 55% (31–71% in uncertainty analysis), as found among older women in a recent trial [19]. Lastly, we assumed injectable ARV agents would be introduced (**phase 3)**.

**Table 1:** Scenarios modelled

	Products	HIV protection	Pregnancy protection	STI protection
Phase 1	Oral PrEP	X		
Phase 2	Oral PrEP	X		
	Vaginal ring	X		
Phase 3	Oral PrEP	X		
	Vaginal ring	X		
	Injectable ARVs	X		
Phase 4	Oral PrEP	X	X^a^	X^a^
	Vaginal ring	X	X^a^	X^a^
	Injectable ARVs	X	X^a^	X^a^

^a^Two scenarios are modelled for Phase 4, one including just pregnancy protection and another including both pregnancy and STI protection

Although injectable ARVs have not yet been evaluated among women in HIV prevention trials, we assumed a higher HIV efficacy of 75% (55–90% in uncertainty analysis) because there should be fewer issues of adherence. For oral PrEP, vaginal ring and injectable ARVs, we initially assume no pregnancy or STI protection (in phase 3), but then assess in **phase 4** how uptake would increase if they also had pregnancy protection or both pregnancy and STI protection with the same efficacy. This was done to reflect current products in development as well as other possible products not yet in development [39].

### Summary of discrete choice experiment

The DCE protocol and results are described elsewhere [40, 41]. In brief, a DCE was developed to elicit stated preferences for new HIV prevention products with their attributes varying as in Table 2. The DCE options were developed through qualitative focus groups, literature review, and extensive piloting. Primary data collection occurred in October to December 2015 in Ekurhuleni (south-east of Johannesburg, South Africa) in a peri-urban area. The study was approved by the University of the Witwatersrand Human Research Ethics Committee and the Research Ethics Committee at the London School of Hygiene and Tropical Medicine. All participation in the DCE, alongside supporting qualitative studies, was voluntary and subject to completion of written informed consent.

**Table 2:** Base case product characteristics. Simulated characteristics in parentheses

Product	HIV efficacy (bounds used in uncertainty analysis – uniform distribution)	STI efficacy	Contraceptive protection	Frequency of use
Oral pre-exposure prophylaxis	61% (40–75%)	N (Y)	N (Y)	Daily
Vaginal Ring	55% (31–71%)	N (Y)	N (Y)	Monthly
Injectable ARVs	75% (55–90%)	N (Y)	N (Y)	Every three months
Condom	85% (66–94%)	Y	Y	Coital
No condom	0	N	N	Coital

Data were gathered through a randomised household survey from 158 adult females (age 18 or over) and 204 adolescent girls (aged 16–17) who self-reported HIV negative status [42]. DCE analyses are based on the assumption that people maximise their utility [31]. By analysing how respondents make choices over the hypothetical choice sets in the DCE, we can infer how important different attributes are to their decision-making. Further information on how this was done can be found in the Supplementary material. This method of simulating from choice data has been termed predicted probability analysis, and has been applied in various fields [43, 44]. We use a nested logit model for prediction, which in part accounts for the assumption of independence of irrelevant alternatives, a limitation of many choice models. In health, there is evidence that hypothetical choices in DCEs correlate with real-world choices [45].

In this analysis, we developed separate uptake projections for adolescent and adult women and considered the following ARV-based prevention products - oral PrEP, vaginal ring and injectable ARV agents. We undertook separate analyses for adolescent and adult women because these subgroups have differing levels of HIV risk [46, 47] and sexual risk behaviours [46], with the adverse effects of these risks being different [48]. We also expected them to have different preferences for sexual and reproductive health products, dissimilar levels of health service utilisation [46], and would need tailored interventions to meet their needs [49]. Uptake projections were produced for women using or not using condoms in their last sex act, which were combined to give the overall degree to which each product would be used. Uptake projections were made for the four phase scenarios described above.

### Estimating prevention protection

We adapt a formula from our previous paper to estimate the short-term impact of a number of HIV prevention products on the average level of protection that a woman has (defined as the prevention protection) [30]. This measure gives the overall average decrease in the probability of HIV transmission in an average sex act resulting from a number of products with different efficacies being used at different levels. For a single product *x*, we assume the average protection against HIV, *P*_*x*_, from using product *x* is the product of its efficacy, *E*_*x*_, and uptake (or use) *U*_*x*_,

1 $$ {P}_x={E}_x{U}_x. $$

In this analysis, we assume *P*_*x*_ is the existing protection provided by male condoms (defined as product x). Our DCE then gives projections of the degree to which condom users and non-condom users uptake each product (from 1 to 3 products), and for prior condom users the degree to which the woman would still use condoms (ε, assumed to be independent of product) in addition to the new product. For *n* new products, each with efficacy *E*_*i*_ and uptake *Uc*_*i*_ and *Unc*_*i*_ (*i = 1..n*) among condom users and non-condom users, respectively, the overall protection provided (*P*_*n*_) is estimated as:

2 $$ {P}_n={U}_x\left[\left(1-\left(1-\varepsilon \right)\sum \limits_{i=1..n}{Uc}_i\right){E}_x+\left(1-\varepsilon \right)\sum \limits_{i=1..n}{E}_i{Uc}_i+\varepsilon \sum \limits_{i=1..n}\left(1-{E}_x\right){E}_i{Uc}_i\right]+\left(1-{U}_x\right)\sum \limits_{i=1..n}{E}_i{Unc}_i $$

It is important to note that the uptake of each product (*Uc*_*i*_ and *Unc*_*i*_) will depend not only on its HIV efficacy but also on whether they provide STI and/or pregnancy protection. The prevention protection provided by different products is assumed to be additive if they are not used together, which was assumed for all ARV-based products. However, when condoms were used with a new product, the new product was assumed to decrease the remaining risk still existing after the protection provided by the condom was accounted for.

The formula in eq. 2 is used to estimate the prevention protection provided in each phase by the available combined prevention products. In each phase, we estimate the degree to which the addition of a new product increases the existing prevention protection among young and adult women in South Africa, and how the non-HIV characteristics of that product improve its HIV effectiveness through increasing uptake. The scenarios modelled are shown in Table 1, using the uptake projections from the DCE and the efficacy estimates in Table 2. Based on data from the DCE, we assumed 43% condom use in last act among adult women and 65% among adolescents at baseline [41]. This is comparable to condom use estimates from the 2017 South African National HIV prevalence, incidence, behaviour and communication survey [47].

### Uncertainty analysis

To assess the robustness of our prevention projections, we undertook an uncertainty analysis to see how uncertainty in the product HIV efficacy (Table 2) and resulting DCE uptake projections (from the 95% confidence intervals for the model) affect the prevention protection achieved in an average sex act, the degree to which each product contributes to the prevention protection, and the importance of pregnancy and STI protection for increasing the protection provided by the products. We considered the scenario where all 3 products have been introduced (phases 3 and 4) with oral PrEP, vaginal ring and injectable ARVs either having no pregnancy or STI protection, just pregnancy protection, or both STI and pregnancy protection. For uptake predictions, Monte Carlo simulation was used with 1000 independent draws from the normal distributions of DCE parameters, simultaneously varying product efficacy according to a uniform distribution between upper and lower bounds in Table 2.

## Results

### Projected uptake of products in adolescent and adult women

**Fig. 2:**
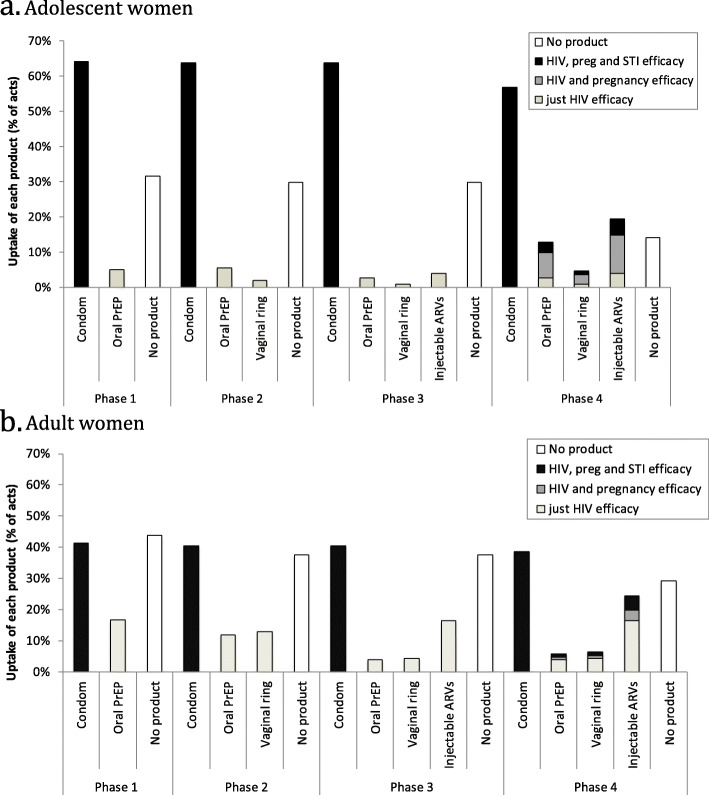
Projected uptake of oral PrEP, vaginal ring and injectable ARVs amongst adolescent (**a**) and adult women (**b**), and resulting effect on levels of condom use. The percentage of women using no product for each phase is also shown. The products are assumed to be introduced in four phases, with oral PrEP becoming available first, then the vaginal ring, and injectable ARVs (these 3 are assumed to be just HIV efficacious), and then phase 4 has the same products but with pregnancy and STI efficacy included. **a**. Adolescent women. **b**. Adult women

The DCE suggests that the patterns of uptake of oral PrEP, vaginal ring, and injectable ARVs will depend on the target population using the products, the availability of other products, and the characteristics of the product (Fig. 2). However, condom use remains fairly stable irrespective of what other products are available, with it only reducing by up to 8 percentage points, from 43 to 39% in adult women and 65 to 57% in adolescents, when all three additional products are available, and they all provide protection against pregnancy and STIs.

Among **adult women**, we predict that 17% of adult women would use oral PrEP if it was the only product available (other than condoms) and was just HIV efficacious (phase 1). However, its use would decrease by a third in phase 2 when the vaginal ring is also available, with both products being similarly popular (used by 12–13% of adult women) if they are just HIV efficacious. In phase 3, the projected uptake of both oral PrEP and vaginal ring decreases by two-thirds (< 4.5% use either) because many (16%) women switch to using injectable ARVs. Lastly, in phase 4, when the different products now provide multipurpose protection, our projections suggest adult women in South Africa will favour injectable ARVs with 20% using this product if it provides pregnancy protection and 24% if it also provides STI protection, while less than 5% use oral PrEP or the vaginal ring.

In contrast to adult women, **adolescent women** have a much stronger preference for products providing pregnancy protection (and STI protection to a smaller extent), with a product’s HIV efficacy being relatively less important. For instance, in phase 4 the uptake of each product increases nearly 4-fold when pregnancy protection is included compared to if the products just provided HIV protection, and increases a further 30% if STI protection is also included. For injectable ARVs this means that uptake increases from 3.9% if they are just HIV efficacious, to 15% if they also provide pregnancy protection and 19% if they provide pregnancy and STI protection. Importantly, there is very little uptake of any product if it is only HIV efficacious with at best 5% uptake being achieved with oral PrEP in phase 1 and 2. In terms of product preferences, adolescent women seem to have little preference for the vaginal ring even when it has pregnancy and STI efficacy. As seen for adult women, injectable ARVs seem to be the most widely preferred product by these populations in South Africa, although oral PrEP is still liked.

Importantly, even when all three new products are available a sizeable proportion of adolescent (14–30%) and adult (29–38%) women are not inclined to use any form of protection, irrespective of whether the products also provide pregnancy and STI protection.

### Projected protection provided in adolescent and adult women

**Fig. 3:**
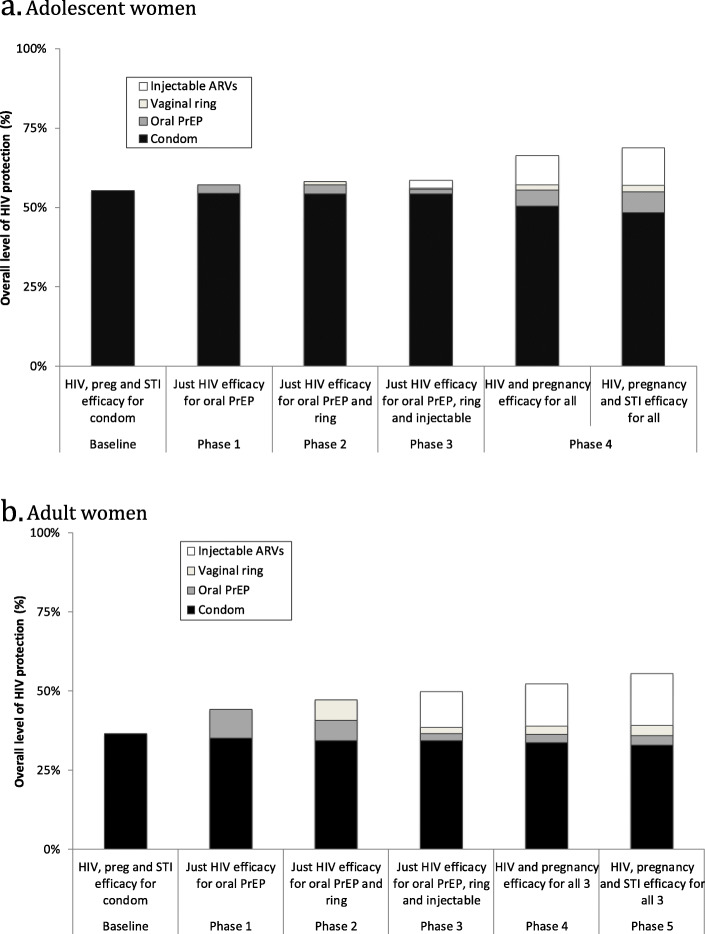
Projected overall protection provided by introducing oral PrEP, vaginal ring and injectable ARVs amongst adolescent (**a**) and adult women (**b**), in addition to baseline levels of condom use. The products are assumed to be introduced in four phases, with oral PrEP becoming available first, then the vaginal ring, and injectable ARVs (these 3 are assumed to be just HIV efficacious), and then phase 4 has the same products but with pregnancy and STI efficacy included. **a**. Adolescent women. **b**. Adult women

When we incorporate the DCE uptake projections into our model, it suggests that the introduction of these products in adolescent women will only contribute markedly to the existing protection supplied by condoms (Fig. 3a) if multiple products are introduced and they also provide protection against pregnancy (and STIs to a lesser extent). For instance, the total additional protection provided by solely HIV efficacious products is at best 3 percentage points, whereas the introduction of multiple products that also have pregnancy protection increases the protection supplied by 11 percentage points (from 55 to 66% in absolute terms) and 14 percentage points (to 69%) if they also protect against STIs.

In contrast, greater benefit can be achieved through introducing solely HIV efficacious products among adult women (Fig. 3b), but less additional impact is achieved if they also incorporate pregnancy and STI protection. For instance, introducing just oral PrEP (phase 1) is projected to increase the protection provided to adult women from 37 to 44% if it is just HIV efficacious; increasing to 47% if the vaginal ring is introduced and 50% if injectable ARVs are also introduced. However, incorporating pregnancy protection to these three products only increases the protection to 52, and 56% if they also protect against STIs.

Importantly, as also highlighted in the uptake projections, our modelling here suggests that even with introduction of three new multi-purpose HIV prevention products, there is likely to remain a large prevention gap with the imperfect use and efficacy of these products resulting in at least 31 and 44% of adolescent and adult women’s baseline transmission risk still remaining, respectively.

### Uncertainty analysis

Consistent with our point projections for adolescent women, our uncertainty analysis found that 59–62% of model runs projected that the addition of pregnancy protection was more important for increasing uptake than HIV efficacy for oral PrEP, vaginal ring and injectable ARVs, with its addition increasing the overall protection provided by these products by 138% (IQR 71–244%) compared to if the products were just HIV efficacious. Additionally, injectable ARVs were the most preferred product for adolescent women in 80% of model runs, while the vaginal ring was least popular in 95% of model runs. Uncertainties in the uptake projections resulted in moderate uncertainty in the overall protection provided by the three products, as can be seen in Supplementary Fig. 1a.

Similarly, the results of our uncertainty analysis for adult women were also generally consistent with our point projections, with HIV efficacy being the most important attribute of a product in 94% of model runs, and injectable ARVs being the most preferred product in 99% of model runs. Supplementary Fig. 1b shows that there was also moderate uncertainty in the protection projections for adult women, but as for the point projections less was gained from incorporating pregnancy and STI efficacy, with the addition of these attributes increasing the protection provided by solely HIV efficacious products by 41% (IQR 26–71%).

## Discussion

Oral PrEP is now available in many settings, while other new ARV based HIV prevention products should become available in the next 5–10 years [17, 18, 25]. With the concurrent scale-up of ART, this has raised optimism that we can reduce HIV transmission to low levels by 2030, as advocated by the World Health Organisation [50]. To achieve this, we need high uptake of new prevention products in the groups with highest burden, such as young and adult women [46, 51]. Although trials and demonstration projects provide insights into how new products may be used [13, 52], they cannot give insights for products that are in earlier stages of development or how including other product characteristics may affect levels of use.

Our analyses help to fill this knowledge gap. They emphasise the importance of multi-purpose prevention products for ensuring high uptake and protection amongst adolescent women, and for improving uptake and protection amongst adult women. Also, adolescents and adult women are likely to need a range of products, with their ultimate preference being towards injectable ARVs, less so oral PrEP and the vaginal ring, and for adolescents any product that provides pregnancy protection. The development of a jointly HIV and pregnancy protective product is critical to meet the needs of adolescent women who are at heightened risk of HIV, since preferences for contraceptive products are particularly great in this group.

Importantly, irrespective of what products are introduced, a large prevention gap (> 20% women remain unprotected) may remain among both groups following the introduction of these new products. This highlights the importance of interventions to encourage greater use of new ARV-based prevention products amongst women; one of the aims of the Dreams intervention for adolescent women in sub-Saharan Africa (https://www.state.gov/pepfar-dreams-partnership/). Possible strategies to encourage greater uptake include demand creation involving positive communication and awareness raising, while greater adherence could be promoted through PrEP adherence support or peer support groups, while integrating PrEP visits with other reproductive health services may promote greater attendance at visits [13].

### Limitations

Our analysis has limitations. Firstly, uptake projections from a DCE are theoretical and not based on actual long-term use of a product. Although real levels of product use are likely to differ from our projections, it is re-assuring that they agree qualitatively with the observed low uptake of oral PrEP and vaginal ring among young women [11, 12, 19, 53], and the high acceptability of injectable contraceptives amongst South African women [12]. They also agree with other studies that have emphasised the preference for multipurpose prevention technologies (MPTs) in women [54–56]. However, it is still important to remember the hypothetical nature of our study, with our uptake projections likely being optimistic compared to real levels of use. This was seen for PrEP, where high levels of acceptability prior to its introduction [57, 58] has not translated into similar levels of use among women [13, 59]. In other health areas, DCEs have been shown to predict opting-in behaviours with a 88% sensitivity indicating that, although imperfect, they can be useful tools when the alternative is to make assumptions without data from end-users [45].

Secondly, it is possible that the high acceptability of injectable ARVs among adult women may not be generalizable to other settings where there has not been the same history of using injectable contraceptives. However, this may not be an issue because other studies have also suggested that injectable ARV may be more acceptable to women because they require less user involvement and could be long-lasting [14, 56].

Thirdly, our impact projections are likely to be conservative because we did not account for the additional benefit that an STI efficacious product may have on HIV transmission through reducing STI transmission. Conversely, we did not account for a possible increase in HIV transmission risk resulting from combining hormonal contraceptives with an ARV-based product, in line with the results of the ECHO trial [60].

### Comparison with other analyses

Other modelling has projected the impact of combination HIV prevention products, using expert opinion and trial data to estimate the likely level of use of new products [25–28]. Our uptake projections are generally consistent or more conservative than were used in these analyses, and differ by population group. These differences highlight that we should be wary of using expert opinion to guide uptake scenarios because they are not based on actual user preferences and could overplay the likely impact of new products. There was further novelty to our analysis because we also assessed the effect on uptake of developing MPT products with pregnancy and STI protection as well as HIV efficacy. This has not been done in previous analyses except our previous cost-effectiveness analysis [36], which showed incorporating pregnancy protection could improve the cost-effectiveness of HIV prevention products.

### Implications and conclusions

High levels of HIV transmission exist among women in sub-Saharan Africa, with existing interventions being insufficient to reduce their heightened risk. It is hoped that newly emerging ARV-based prevention products may fill this prevention gap. Our projections suggest this could be the case, but only if these new products have multiple facets for adolescent women, incorporating pregnancy and STI protection for maximising their uptake. Indeed, a product’s non-HIV characteristics may be more important for adolescent women than its HIV efficacy, with little uptake and impact being achieved otherwise. This is less important for adult women where HIV efficacy is more important. It is uncertain why adult women do not view STI protection as importantly as HIV protection when choosing between products, although it may be partly due to having less knowledge about STIs [61] and/or HIV being seen as a more serious disease than other STIs. It is also important that the full range of products are available to women for maximising uptake, with injectable ARV agents probably being most popular for adolescent and adult women. Importantly, though, our projections suggest that in neither adolescent nor adult women will the introduction of these products result in the overall average protection being higher than 60%. This means that a substantial portion of acquisition risk will still exist, emphasising the need for interventions to increase uptake of new products and the importance of scaling up HIV treatment.

## Supplementary information

**Additional file 1.**

## Data Availability

The study data is available from Matthew Quaife and the model is available from Peter Vickerman.

## Abbreviations

ART: Antiretroviral therapy
HPTN: HIV Prevention Trials Network
HIV: Human immunodeficiency virus
PrEP: HIV pre-exposure prophylaxis
ARV: Antiretroviral
STI: Sexually transmitted infection
DCE: Discrete choice experiment
IQR: Inter Quartile range
MPT: Multipurpose prevention technologies
