# Supplementary material for: HIV prevention is not all about HIV – using a discrete choice experiment among women to model how the uptake and effectiveness of HIV prevention products may also rely on pregnancy and STI protection
Source: BMC Infect Dis. 2020 Sep 25;20:704. doi: 10.1186/s12879-020-05399-4 (PMC7517801; doi:10.1186/s12879-020-05399-4)
Supplement: Supplementary file 1 — Additional file 1. [file 12879_2020_5399_MOESM1_ESM.docx]

**Supplementary material: The Nested Logit Model**

Uptake predictions were generated in a discrete choice experiment (DCE) analysis, conducted by some of the study authors in 2015([41](#_ENREF_41)). A nested logit model was used to analyse DCE data, which has the advantage of avoiding the independence of irrelevant alternatives assumption required in the more widely-used multinomial logit model. Although this allows for more realistic substitution patterns between the choice to use any products or no products, predictions between products may still suffer from this. We do not rescale any parameters due to the absence of revealed preference data for new products among these groups. The theory of choice modelling behind the nested logit model follows.

If we start from the utility function for a choice alternative, *i*, based on random utility theory, where utility, *U*, is made up of a systematic component, *V*, and a random component, ε, representing unobserved differences in tastes: $U_{i} =V_{i} +\varepsilon_{i}$. *V* is the sum of individual *q*’s objective utilities, *ß,* of the service/benefit set of attributes of *i* with attributes *k* and the subjective utilities, *X,* of consuming *ik*: $\text{V}_{\text{iq}}=\sum_{k=1}^{K} \beta_{ik}X_{ikq}$([43](#_ENREF_43)).

The nested logit model allows the variance of $\varepsilon_{i}$to differ between the branches (nests), but not within. This means that $\varepsilon_{i}$is the sum of the unobservable utility at the branch level $\varepsilon_{j}$and at the elemental alternatives level $\varepsilon_{k\left|j\right.}$. The utilities are then modelled separately at the different levels and can be partitioned into two choices: 1. Participate, Not participate, with an associated utility $U_{j}$; and 2. If participate, the choice is between the new prevention technology alternative, and their associated utilities $U_{k\left|j\right.}$.

$\varepsilon(j,k)=\varepsilon_{j}+\varepsilon_{k\left|j\right.}$ , $U(j,k)=U_{j}+U_{k\left|j\right.}$ and $V(j,k)=V_{j}+V_{k\left|j\right.}$

The unconditional probability of choosing a specific alternative $U_{j,k}$is the sum of the marginal probability at the branch level $P_{j}$and the conditional probability at the elemental alternative level, $P_{k\left|j\right.}$[[1]](#footnote-1).

$$P(j,k)=P_{j}+P_{k\left|j\right.}$$

The scale parameter is the part of the variance of the unobserved utility that is allowed to vary between nests (for more detail, see ([43](#_ENREF_43)) or ([63](#_ENREF_63))), and provides the link between these levels. The inclusive value is the ratio of the scale parameters of the upper $\lambda_{j}$ to the lower level$\mu_{k}$.

$$IV=\frac{\lambda_{j}}{\mu_{k}}$$

For identification purposes, one of the scale parameters must be normalised to 1, this is usually done at the upper (branch) level. The inclusive value parameter provides the basis for testing the appropriateness of the nested logit model. The closer it is to 1, the closer the levels are to having the same variance in their error terms: if it is equal to 1, the model collapses to a multinomial logit model. The closer it is to 0 the greater the perceived similarities between the alternatives within the nest.

Participate

Not participate (C)

A

B

Branches *j*

Elemental alternatives *k*

Figure S1: The nested logit tree structure

1. Henscher D, Rose J, Greene W (2005) Applied Choice Analysis: A Primer. Cambridge: Cambridge University Press.

2. Brau R, Bruni ML (2008) Eliciting the demand for long-term care coverage: a discrete choice modelling analysis. Health Econ 17: 411-433.

**Supplementary figure 1**: Uncertainty (median and interquartile range) in the total protection provided in phase 4 for adolescent and adult women depending on whether the products also have pregnancy, pregnancy and STI protection, or neither.

4a Adolescent women

4b Adult women

1. Calculation of probabilities from utility parameters is not as straightforward in the nested logit model. As they entail extensive equations it has been chosen not to present them or calculate them. Instead probabilities are obtained using the *Prob* command in NLOGIT software. The predicted shares are also obtained from NLOGIT’s simulation command. [↑](#footnote-ref-1)
